# Invasive alien shredders clear up invasive alien leaf litter

**DOI:** 10.1002/ece3.4430

**Published:** 2018-10-03

**Authors:** Thomas M. Doherty‐Bone, Alison M. Dunn, Joel Brittain, Lee Eric Brown

**Affiliations:** ^1^ School of Geography University of Leeds Leeds UK; ^2^ School of Biology University of Leeds Leeds UK; ^3^ Water@Leeds University of Leeds Leeds UK

**Keywords:** ecosystem functioning, *Eriocheir sinensis*, *Pacifastacus leniusculus*, *Rhododendron ponticum*

## Abstract

Biological invasions have the potential to alter ecosystem processes profoundly, but invaders are rarely found alone. Interactions between different invasive alien species, and their cumulative impact on ecosystem functioning, have led to hypotheses of invasion meltdown whereby effects become additive leading to further ecosystem stress. Invasive riparian plants (e.g., *Rhododendron ponticum*) deposit leaf litter in freshwaters, which may be unconsumed by indigenous species, potentially affecting habitat heterogeneity and flow of energy to the food web. However, invasive alien decapod crustaceans are effective consumers of leaf litter, and it was hypothesized that they would also consume inputs of invasive riparian leaf litter. This study shows that invasive alien signal crayfish (*Pacifastacus leniusculus*) and Chinese mitten crab (*Eriocheir sinensis*) effectively break down different types of leaf litter, including invasive alien *R. ponticum,* at higher rates than indigenous white‐clawed crayfish. Secondary products were more varied, with more fine particulate organic matter generated for the less palatable alien leaf litter species. Leaf species caused different changes in body mass of decapods but effects were heterogeneous by leaf and decapod: *P. leniusculus* showed lower mass loss when consuming *R. ponticum* while *E. sinensis* lost mass when consuming *A. pseudoplatanus*. Impacts of riparian invasions on detritus accumulation in freshwaters are thus potentially buffered by invasive alien decapods, illustrating a need for a more detailed consideration of both positive and negative interspecific feedbacks during biological invasions.

## 1 INTRODUCTION

Invasive alien species (IAS) threaten global biodiversity, ecosystems, and economies, occurring at many trophic levels simultaneously (Simberloff et al., 2013). Many invasive species co‐occur, leading to the hypothesis of invasive meltdown whereby effects multiply to cause more substantial issues than if invaders were found alone, as with other multiple stressors (Jackson, Loewen, Vinebrooke, & Chimimba, 2016). However, studies on the combined impacts of multiple invasive species on ecosystem functioning are limited and mostly restricted to between‐species interactions with invasion success the primary outcome measured. Interactions between IAS may be mutually facilitative (“invasion meltdown,” Simberloff & Von Holle, 1999), antagonistic, or neutral (Jackson, 2015). For example, one IAS might consume another, but also consume its competitors, with both increasing in abundance (zu Ermgassen & Aldridge, 2011). Two IAS might consume resources complementarily to result in synergistic resource depletion (Rosewarne et al., 2016). The potential impact of these interactions with multiple IAS on ecosystem functioning has so far been little studied.

In freshwater ecosystems, two prominent IAS guilds are riparian plants and omnivorous animals (Gallardo, Clavero, Sánchez, & Vilà, 2015). Invasive alien riparian plants affect freshwater ecosystem processes through reducing light levels and by introducing allochthonous leaf litter that could be novel to the ecosystem either in quantity or in quality (Hladyz, Åbjörnsson, Giller, & Woodward, 2011; Hladyz, Gessner, Giller, Pozo, & Woodward, 2009). Invasive alien omnivorous animals (e.g., amphipods, decapods) affect freshwater ecosystems through the direct consumption (shredding) of detritus, as well as trophic cascades following consumption of detritivores and ecosystem engineering (Doherty‐Bone, Dunn, Liddell, & Brown, 2018; Gallardo et al., 2015; Harvey et al., 2013). These two guilds of IAS (riparian plants, shredders) when considered in isolation have the potential to impact detrital processing in freshwater ecosystems. Leaf litter is converted into smaller fragments by shredding animals, either mechanically through tearing up of the leaf material or digestively through passage through the gut (Doherty‐Bone et al., 2018; Montemarano, Kershner, & Leff, 2007). Studies of invasive alien leaf litter have shown both enhanced and reduced decomposition rates by indigenous consumers (freshwater microbes, invertebrates) (Hladyz et al., 2009). Invasive alien omnivores have been found to increase decomposition rates relative to native analogues (Doherty‐Bone et al., 2018; Dunoyer, Dijoux, Bollache, & Lagrue, 2014; James, Slater, Vaughan, Young, & Cable, 2015). Yet, the combined effects of invasive riparian plants and invasive alien omnivores on detrital processes have not been investigated.

**Figure 1:**
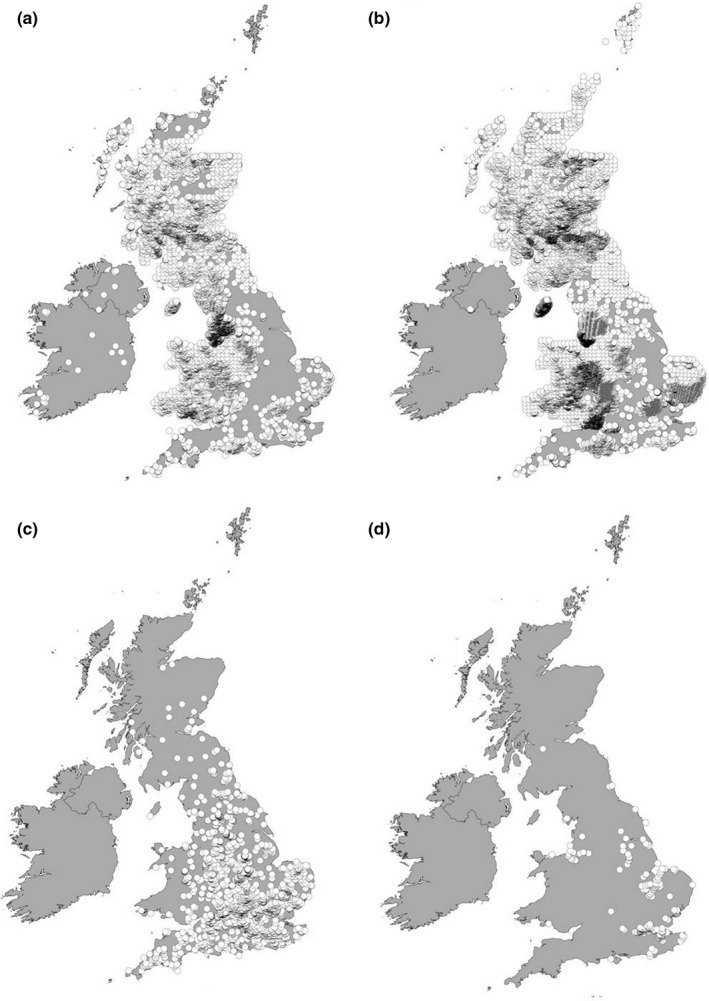
Map showing overlap in distribution in the British Isles of invasive alien riparian plants (a) *Rhododendron ponticum*, (b) Sycamore (*Acer pseudoplatanus*) and the invasive alien decapods (c) American Signal Crayfish (*Pacifastacus leniusculus*), and d) the Chinese Mitten Crab (*Eriocheir sinensis*). Source: National Biodiversity Network Atlas http://www.nbnatlas.org Accessed 01 April 2018 (see Supporting information Appendix S1 for details of distributional data sources)

This study compares the processing of leaf litter from invasive riparian woody plant species by invasive freshwater decapods and contrasts these to equivalent native species. The study focused on two prominent invasive alien plants in the British Isles, *Rhodendendron ponticum* Linnaeus and *Acer pseudoplatanus* Linnaeus. Both these species were introduced from mainland Europe and are now widespread in the British Isles (Figure 1a), as well as other regions such as Scandinavia and New Zealand, where they are known to become dominant, shade out other plants and waterways, as well as the leaf litter being of comparable lower quality (Hill & Hulme, 2012; Hladyz et al., 2009, 2011; Squirrel, 2015). The invasive alien decapod species included in this study are *Pacifastacus leniusculus* Dana 1852 (the American Signal Crayfish) originating from the North West of North America and *Eriocheir sinensis* H. Milne‐Edwards 1853 (the Chinese Mitten Crab) originating from Korea and eastern China. Both these decapod species are widespread IAS in the British Isles (Figure 1b), mainland Europe and North America and are rapidly expanding their range (Herborg, Rudnick, Siliang, Lodge, & Macisaac, 2007), likely impacting ecosystems through strong top‐down regulation of smaller animals, plants, and detritus (Rosewarne et al., 2016; Rudnick & Resh, 2005). It was hypothesized that (H_1_) invasive alien decapods would have a higher rate of detrital processing, than native invertebrate shredders as observed by Doherty‐Bone et al. (2018); (H_2_) decomposition rates would be lower for the IAS *R. ponticum* and *A. pseudoplatanus* due to higher tannin and cellulose content than the dominant native riparian species *Alnus glutinosa* (Hladyz et al., 2009). As a consequence, (H_3_) the assimilation (measured by gain in mass) of invasive alien leaf litter will be higher for invasive alien decapods compared to the native crayfish.

## 2 MATERIALS AND METHODS

We compared processing of detritus from riparian plants of a dominant UK riparian native plant (Black Alder, *A. glutinosa*) to the invasive alien *A. pseudoplatanus* and *R. ponticum* by one native (*Austropotamobius pallipes*) and two invasive (*P. leniusculus, E. sinensis*) decapods. For the three plants, leaf litter was collected upon abscission around the University of Leeds, dried at 50°C for 24 hr then stored in cool, dry, paper bags in the dark. While oven drying can cause plant material to leach mass at a faster, exaggerated rate to leaf litter naturally deposited into waterways (Gessner, Chauvet, & Dobson, 1999), this process enables more accurate comparisons of biomass before and after the experiment. Fourteen days prior to the experiment, 1.5 g leaf was placed in mesh bags and microbially conditioned with water from a nearby stream (Doherty‐Bone et al., 2018). *Austropotamobius pallipes* were collected from Wyke Beck, Leeds, UK, *P. leniusculus* from Fenay Beck, Huddersfield, and *E. sinensis* collected from the River Thames, Chiswick. Decapods were kept in aged tap water for a minimum of 14 days and unfed for 24 hr hours prior to use in the experiment.

Each microcosm consisted of a 4‐L plastic tank with aerated, aged tap water with a PVC pipe shelter for the decapods. A base of nylon mesh (1 mm aperture) allowed organic matter <1 mm to fall through to a separate chamber (Doherty‐Bone et al., 2018). These were maintained in an environmental chamber at 14°C on a 16:8 hr light:dark cycle throughout the experiment.

### 2.1 Experimental design and sample processing

Twelve treatments (10 replicates/treatment, 120 microcosms total) were established containing the conditioned leaf litter from one of the three plant species, and a decapod treatment (native crayfish, invasive crayfish, invasive crab, control without decapod). Subadults of similar sizes were used because they are the most common age class encountered in the field (Doherty‐Bone et al., 2018). At the start of the experiment, decapods were weighed following being dabbed dry with a paper towel: *A. pallipes –* 12.87 ± 2.92 g; *P. leniusculus* – 10.33 ± 2.98 g; *E. sinensis* – 11.02 ± 4.28 g. The experiment ran for 7 days. At the end of the experiment, decapods were weighed to determine changes in mass after being dabbed dry with a paper towel. Remaining leaf litter and smaller leaf fragments (coarse particulate organic matter [CPOM]‐ 10‐1 mm sized fragments) were collected. The remaining water was homogenized and a 50 ml aliquot removed to sample fine particulate organic matter (FPOM – 1 mm–0.7 μm) that was then filtered through 0.7 μm GF/F filters. Samples of leaf litter, CPOM, and FPOM were dried separately at 50°C, weighed and ashed at 500°C to estimate ash‐free dry mass (AFDM).

### 2.2 Data analysis

Decomposition rate was calculated as the change in AFDM of leaf litter per day, final AFDM estimated based on the baseline AFDM estimated from a separate batch of conditioned leaf litter of each species (Benfield, 2006). As mass of a decapod can influence consumption rates of leaf litter, as well as to enable estimation of impact by mass of animal, rates of decomposition and production of residual materials were divided by decapod mass to show mass‐specific performance (Doherty‐Bone et al., 2018). This enabled measurement of both the actual impact (decomposition rate, etc.) and the quantitative functional trait of detritivory performance (decomposition rate per mass of animal). Generalized linear models (formula: glm, R v.3.1.0.; R Development Core Team, 2014) were used to compare decomposition rate, CPOM and FPOM production, and change in decapod mass against leaf and decapod species treatment, with decapod mass included as a covariate. The models were fitted based on the distribution of the data (i.e., family = Gaussian; Poisson) identified using maximum likelihood estimates (library: MASS, formula: fitdistr). Model fit of the glm was compared using Akaike information criterion (AIC) (Burnham & Anderson, 2004) and Tukey post hoc tests applied to establish pairwise differences (library: mcp, formula: glht, tables S 1‐5, Bretz, Hothorn, & Westfall, 2010).

## 3 RESULTS

**Figure 2:**
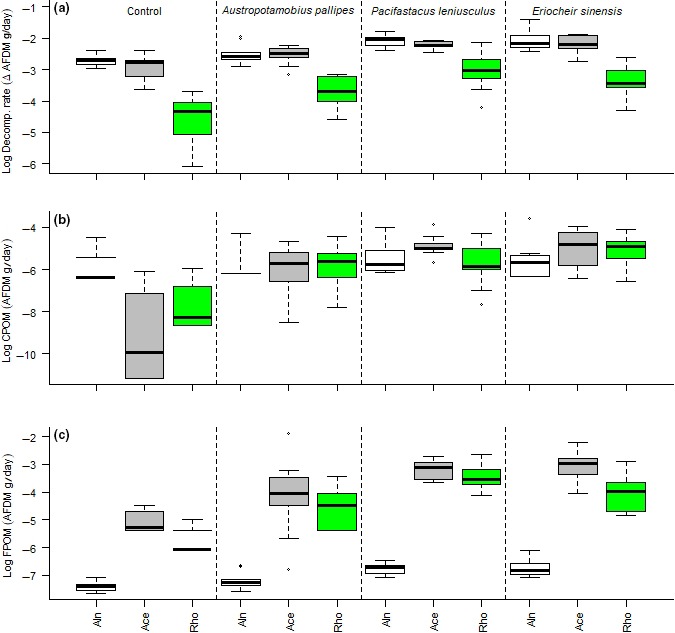
Decapod (species shown at top) processing of detritus in relation to leaf species, showing (a) decomposition rates, (b) CPOM production, and (c) FPOM production for *Alnus glutinosa* (white bars), *Acer pseudoplatanus* (gray bars), and *Rhododendron ponticum* leaves (green bars). The *y*‐axis is log‐transformed (but not the data analysis) to aid visualization

Both leaf species and decapod species had a significant effect on leaf decomposition rate and production of FPOM, the interaction between leaf and decapod species showing the highest performing models (ΔAIC = 0) (Table 1). Production of smaller fragments of CPOM was affected by decapod species but not by leaf species (Table 1). Decomposition rates were similar between the invasive alien *A. pseudoplatanus* and the native *A. glutinosa* but lowest for *R. ponticum* (Figure 2a, Supporting information Table S1). There were significant interactions between leaf species, decapod species, and decapod mass for decomposition rate (albeit with a low likelihood based on a high ΔAIC, Table 1), with higher decomposition rates for the invasive alien *P. leniusculus* and *E. sinensis* than for the native *A. pallipes* (Figure 2a). Notably, when compared with controls and the native *A. pallipes* treatment, *R. ponticum* leaf litter decomposed faster in the invasive *P. leniusculus* and *E. sinensis* treatments.

**Table 1:** Generalized linear models of variables for detrital processing. Mass refers to the wet mass of the decapod. Values in bold show *p*‐values <0.05

Response variable	Model	df	Residual deviance	Pr (>Chi)	AIC	ΔAIC
Decomposition	Decapod Sp.	3	0.057	**<0.001**	−425	105
	Leaf Sp.	2	0.112	**<0.001**	−469	69
	Decapod * Leaf	6	0.006	0.123	−530	0
	Decapod * Mass	2	0.015	**0.014**	−306	224
	Leaf * Mass	2	0.000	0.878	−356	174
	Decapod * Leaf * Mass	4	0.011	**<0.001**	−394	136
CPOM	Decapod Sp.	3	0.000	**<0.001**	−942	0
	Leaf Sp.	2	0.000	0.328	−918	24
	Decapod * Leaf	6	0.000	0.348	−937	5
	Decapod * Mass	2	0.000	0.889	−682	260
	Leaf * Mass	2	0.000	0.948	−−676	266
	Decapod * Leaf * Mass	2	0.000	0.399	−670	272
FPOM	Decapod Sp.	3	0.011	**<0.001**	−562	62
	Leaf Sp.	2	0.021	**<0.001**	−586	38
	Decapod * Leaf	6	0.007	**<0.001**	−624	0
	Decapod * Mass	2	0.011	**<0.001**	−407	217
	Leaf * Mass	2	0.000	0.588	−437	187
	Decapod * Leaf * Mass	4	0.001	0.536	−430	194
∆Decapod mass	Decapod Sp.	2	28.260	0.343	152	10
	Leaf Sp.	2	25.658	**0.016**	146	4
	Decapod * Leaf	4	21.065	**0.008**	142	0

Invasive alien decapods produced more residual CPOM (a likely by‐product of uneaten leaf litter that is difficult to handle and be consumed by larger animals) than *A. pallipes* and controls, but did not differ significantly across leaf species (Figure 2b). FPOM was significantly influenced by the interaction between decapod and leaf species (ΔAIC = 0, Table 1). For all leaf species, significantly more FPOM was produced by invasive alien decapod species than by *A. pallipes* and controls (Figure 2c). *A. glutinosa* leaves resulted in significantly less FPOM production compared to *A. pseudoplatanus* and *R. ponticum* (Figure 2c). Mass‐specific variables such as decomposition rate, CPOM, and FPOM production expressed as change in mass per decapod body mass, all showed significant relationships to decapod and leaf species. These showed the same relationships as the actual decomposition rates, CPOM, and FPOM production: invasive alien decapods showing higher mean values (Figure 2). Efficiency of leaf litter decomposition showed the best fitting model for leaf and decapod species, as did CPOM and FPOM production efficiency (Table 2).

**Table 2:** Generalized linear models for decapod performance (mass specific) for detrital processing. Values in bold show *p*‐values <0.01

Response variable	Model	df	Residual deviance	Pr (>Chi)	AIC	ΔAIC
Decomposition efficiency	Decapod Sp.	2	0.000	**<0.001**	−671	94
	Leaf Sp.	2	0.145	**<0.001**	−693	72
	Decapod * Leaf	4	0.000	**<0.001**	−765	0
CPOM efficiency	Decapod Sp.	2	0.000	**<0.001**	−1,109	0
	Leaf Sp.	2	0.000	0.264	−1,098	11
	Decapod * Leaf	4	0.000	0.355	−1,104	5
FPOM efficiency	Decapod Sp.	2	0.000	**0.032**	−812	40
	Leaf Sp.	2	0.000	**<0.001**	−844	8
	Decapod * Leaf	4	0.000	0.089	−852	0

**Figure 3:**
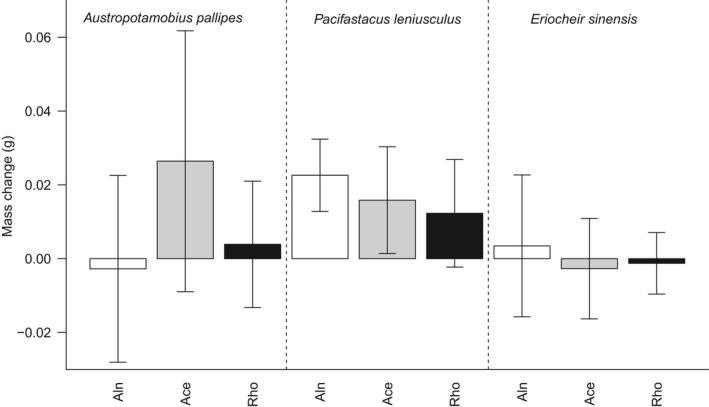
Change in mass of native and invasive alien decapods in relation to leaf species consumed. Bar coloration for leaf species as for Figure 2

The change in decapod mass at the end of the experiment did not differ overall between decapod species, but was significantly affected by leaf species treatment and the interaction between decapod and leaf species (Table 1). The interaction between leaf and decapod species provided the best performing model (ΔAIC = 0). *Post hoc* tests showed heterogeneous mass change in relation to leaf type among decapod species. There was significantly greater mass gain by *P. leniusculus* through the consumption of *A. glutinosa* and *A. pseudoplatanus* leaves and to a lesser degree *R. ponticum* (Figure 3, Supporting information Table S4). In contrast, the native crayfish showed no change for *A. glutinosa* or *R. ponticum*, but showed growth in the presence of *A. pseudoplatanus* leaves. *Eriocheir sinensis* showed little mass change for all three leaf species, though with significant, however marginal body mass loss for *A. pseudoplatanus* compared to *R. ponticum*.

## 4 DISCUSSION

This study provides new insights into the impacts of invasive decapods on invasive alien leaf litter and vice versa. These findings are significant because all four of these IAS are expanding their range in the same regions, often co‐occurring, yet little is known about the consequences of these multi‐invasions. Invasive alien decapods (*P. leniusculus* and *E. sinensis*) processed all species of leaf litter at a faster rate than did the native analogue (*A. pallipes*). The invasive decapods showed higher decomposition rates, converting leaf mass into smaller fragments, and most notably into FPOM. They also processed more of the invasive *R. ponticum* than did the native decapod.

FPOM production was significantly lower for the native leaf *A. glutinosa* and growth of the two invasive alien decapods was either higher or stable, suggesting greater assimilation of the native leaf litter. In contrast, greater FPOM production in the *A. pseudoplatanus* and *R. ponticum* treatments probably reflects undigested leaf material passed through the gut. These invasive plants have a higher cellulose and tannin content and their leaf litter has previously been found to support lower fungal biomass and fewer macroinvertebrates in streams (Hladyz et al., 2009), indicating a likely reduced ability for crustaceans to digest leaf material in the absence of priming by fungal hyphomycetes (Jabiol & Chauvet, 2012). The comparatively low FPOM produced from *A. glutinosa* in comparison with the other two leaf species probably reflects a greater digestion of leaf material by decapods, facilitated by conditioning by hyphomycetes. Given the short time period of the experiment, the relatively small change in mass for the various decapod species is notable, despite the potential for errors through differences in water content. This relationship in mass change and assimilation of leaf litter is however likely to be different in more natural conditions with the presence of invertebrate prey species.

This study provides a novel example of one IAS providing biotic resistance to the effects of another on a key ecosystem process. *Rhododendron ponticum* causes the build‐up of leaf litter in the benthos that is not nutritionally available to microbes or invertebrates, causing shift in benthic food webs and habitat structure in the benthos (Hladyz et al., 2011). While *A. pseudoplatanus* also provides a substantial amount of leaf litter, this is more palatable to aquatic organisms, thus *R. ponticum* by comparison is substantially more impactful (Hladyz et al., 2009). Although the invasive decapods processed more *R. ponticum*, there was negligible growth of decapods consuming *R. ponticum*, suggesting there would be no selective advantage to consuming this material when more nutritious resources are available. In contrast, *A. pseudoplatanus* litter has similar impacts (decomposition rates, N:P, fungal biomass, invertebrate colonization) to other native species, particularly *A. glutinosa* (Hladyz et al., 2009). Testing this interaction in a more realistic mesocosm or in situ cage experiment with multiple available resources would help resolve whether this holds true for real‐world invasion scenarios.

Biological resistance to the establishment of newly colonizing species, including IAS, has been a commonly measured variable of ecosystem functioning (Fargione & Tilman, 2005). Native species providing resistance to the *impacts* of IAS have not been explicitly studied. This is pertinent for native species with high risk of extinction and low ecological redundancy, but with non‐native species replacing their role, as for freshwater decapods in most regions (Lodge et al., 2012). *Austropotamobius pallipes* processed *A. pseudoplatanus* litter showing some biotic resistance to this invasive leaf litter. However, the lack of processing of *R. ponticum* shows the native decapod does not provide a functional resistance to the impacts of this invasive alien shrub, as opposed to invasive alien decapods. Because *A. pallipes* does not consume *R. ponticum* leaves, it is unlikely to be negatively affected through dilution of nutrition. However, *A. pallipes* is likely to suffer from reduced food availability where *R. ponticum* has replaced other riparian plants (Hladyz et al., 2011; Maclean et al., 2017) and could also be impacted by depletion of invertebrate prey if they are excluded by the low nutritional value of the leaf litter.

Potential management applications of this study include prioritizing interventions in waterways selected for staggered removals of first invasive alien riparian plants, then decapods. This would result in processing of the invasive alien leaf litter by the decapods as the source of the litter is removed. As both *P. leniusculus* and *E. sinensis* were also found to maintain, even increase body condition through consuming this invasive alien leaf litter, removal of this riparian resource could potentially, temporarily slow population growth of the invasive alien decapods. Similar recommendations have been made for removal of invasive alien predators after their more impactful prey (Miyake & Miyashita, 2011). However, given the severe impacts and difficulty of control of these invasive alien decapods, rapid response to early introductions of invasive alien decapods should be higher priority.

Invasive alien decapods were shown to remove invasive alien leaf litter, reducing the negative impact of that leaf litter through provision of its otherwise unavailable resources to the remainder of the food web. This provides a clear example of IAS introducing complementarity to biodiversity–ecosystem relationships: Here addition of IAS decapods increased transformation of resources provided by native and alien plants, whereby addition of a species increases a measured ecosystem process such as transformation and capture of a resource (Cardinale, Palmer, & Collins, 2002). The observed interaction of multiple IAS led to a combined, potentially buffering effect on ecosystem functioning.

## CONFLICT OF INTEREST

The authors declare no conflict of interests.

## AUTHOR CONTRIBUTIONS

TDB, AMD, and LEB conceived the study. TDB and JB carried out the experiments. TDB, AMB, JB, and LEB analyzed the data. TDB, AMD, JB, and LEB wrote the manuscript.

## DATA ACCESSIBILITY

https://doi.org/10.5518/208.
