# Supplementary material for: Invasive alien shredders clear up invasive alien leaf litter
Source: Ecol Evol. 2018 Oct 3;8(20):10049–56. doi: 10.1002/ece3.4430 (PMC6206196; doi:10.1002/ece3.4430)
Supplement: Supplementary file 1 [file ECE3-8-10049-s001.docx]

| Supplementary Table 1. Post hoc Tukey test of decomposition rates by decapod species across leaf litter species | | | | |
| --- | --- | --- | --- | --- |
|  | Control | Ap | Pl |  |
| a) Alder |  |  |  |  |
| Control | - |  |  |  |
| Ap | 0.349 | - |  |  |
| Pl | **<0.001** | **0.015** | - |  |
| Es | **<0.001** | **0.002** | 0.924 |  |
|  |  |  |  |  |
|  |  |  |  |  |
| b) Sycamore |  |  |  |  |
|  | Control | Ap | Pl |  |
| Control | - |  |  |  |
| Ap | **0.007** | - |  |  |
| Pl | **<0.001** | **<0.001** | - |  |
| Es | **<0.001** | **<0.001** | 0.999 |  |
|  |  |  |  |  |
| c) Rhododendron |  |  |  |  |
|  | Control | Ap | Pl |  |
| Control | - |  |  |  |
| Ap | 0.107 | - |  |  |
| Pl | **<0.001** | **0.004** | - |  |
| Es | **0.001** | 0.382 | 0.247 |  |
|  |  |  |  |  |

| Supplementary Table 2. Post hoc Tukey test of CPOM production by decapod species across leaf litter species | | | | |
| --- | --- | --- | --- | --- |
|  | Control | Ap | Pl |  |
| a) Alder |  |  |  |  |
| Control | - |  |  |  |
| Ap | 0.964 | - |  |  |
| Pl | 0.805 | 0.555 | - |  |
| Es | 0.730 | 0.480 | 0.999 |  |
|  |  |  |  |  |
| b) Sycamore |  |  |  |  |
|  |  |  |  |  |
| Control | - |  |  |  |
| Ap | 0.368 | - |  |  |
| Pl | **0.003** | 0.076 | - |  |
| Es | **<0.001** | **0.011** | 0.854 |  |
|  |  |  |  |  |
| c) Rhododendron |  |  |  |  |
|  |  |  |  |  |
| Control | - |  |  |  |
| Ap | **0.049** | - |  |  |
| Pl | **0.014** | 0.884 | - |  |
| Es | **0.001** | 0.197 | 0.568 |  |
|  |  |  |  |  |

| Supplemental Table 3. Post hoc Tukey test of FPOM production by decapod species across leaf litter species | | | | |
| --- | --- | --- | --- | --- |
|  | Control | Ap | Pl |  |
| a) Alder |  |  |  |  |
| Control | - |  |  |  |
| Ap | 0.349 | - |  |  |
| Pl | **< 0.001** | **0.015** | - |  |
| Es | **< 0.001** | **0.002** | 0.924 |  |
|  |  |  |  |  |
|  |  |  |  |  |
| b) Sycamore |  |  |  |  |
|  | Control | Ap | Pl |  |
| Control | - |  |  |  |
| Ap | **0.006** | - |  |  |
| Pl | **<0.001** | **0.010** | - |  |
| Es | **<0.001** | **0.014** | 0.998 |  |
|  |  |  |  |  |
| c) Rhododendron |  |  |  |  |
|  | Control | Ap | Pl |  |
| Control | - |  |  |  |
| Ap | **0.025** | - |  |  |
| Pl | **<0.001** | **0.002** | - |  |
| Es | **0.001** | 0.369 | 0.121 |  |
|  |  |  |  |  |

|  |  |  |  |
| --- | --- | --- | --- |
| Supplementary Table 4. Post hoc Tukey test for change in decapod mass across different leaf species | | | |
| Decapod sp. |  | Alder | Sycamore |
|  |  |  |  |
| *A. pallipes* | Alder | - |  |
|  | Sycamore | **0.006** | - |
|  | Rhododendron | 0.684 | 0.102 |
|  |  |  |  |
| *P. leniusculus* | Alder | - |  |
|  | Sycamore | 0.411 | - |
|  | Rhododendron | **0.033** | 0.394 |
|  |  |  |  |
| *E. sinensis* | Alder | **-** |  |
|  | Sycamore | 0.161 | - |
|  | Rhododendron | 0.597 | **0.020** |
|  |  |  |  |

Appendix S1. Sources of occurrence data used to export distribution maps of riparian and decapod species from the National Biodiversity Network Atlas, http://nbnatlas.org

Aggregate Industries

Biological Records Centre

BIS for Powys & Brecon Beacons National Park

Botanical Society of Britain & Ireland

Bristol Regional Environmental Records Centre

Cambridgeshire & Peterborough Environmental Records Centre

Cofnod – North Wales Environmental Information Service

Cumbria Biodiversity Data Centre

Derbyshire Biological Records Centre

Derbyshire Wildlife Trust

Dorset Environmental Records Centre

East Ayrshire Countryside Ranger Service

Environment Agency

Environmental Records Information Centre North East

Essex Wildlife Trust Biological Records Centre

Fife Nature Records Centre

Gloucestershire Centre for Environmental Records

Greater Manchester Ecology Unit

Hertfordshire Natural History Society Flora Group

Highland Biological Recording Group

Isle of Wight Local Records Centre

John Muir Trust

Joint Nature Conservation Committee

Kent Wildlife Trust

Lancashire Environment Record Network

Leicestershire and Rutland Environmental Records Centre

Lorn Natural History Group

Malcolm Storey

Manx Biological Recording Partnership

Marine Biological Association: 295 records

Merseyside BioBank

Ministry of Justice

National Plant Monitoring Scheme

National Trust for Scotland

National Trust

Natural Apptitude

Natural England

Natural Resources Wales

NatureSpot

Norfolk Biodiversity Information Service

North Ayrshire Countryside Ranger Service

Nottinghamshire Biological and Geological Records Centre

Outer Hebrides Biological Recording

Plantlife

rECOrd

Rotherham Biological Records Centre

Royal Horticultural Society

Scotland's Environment Web

Scottish Natural Heritage

Scottish Wildlife Trust

Shire Group of Internal Drainage Boards

Shropshire Ecological Data Network

South East Wales Biodiversity Records Centre

Staffordshire Ecological Record

Suffolk Biodiversity Information Service

Sussex Biodiversity Record Centre

The British Association for Shooting and Conservation

The Conservation Volunteers Scotland

The Wildlife Information Centre

Welsh Government

West Wales Biodiversity Information Centre

Yorkshire Wildlife Trust
